# Classification and diagnosis of the inherited neuropathies

**DOI:** 10.4103/0972-2327.53075

**Published:** 2009

**Authors:** Mary M. Reilly

**Affiliations:** Department of Molecular Neurosciences, MRC Centre for Neuromuscular Disease, National Hospital for Neurology and Neurosurgery and Institute of Neurology, Queen Square, London WC1N 3BG, UK

## Introduction

The inherited neuropathies are a clinically and genetically heterogeneous group of neuropathies in which an accurate genetic diagnosis is increasingly becoming possible. The identification of more than 30 causative genes for the inherited neuropathies has raised important questions regarding the approach to their diagnosis. The move towards developing gene-specific therapies will make accurate genetic diagnoses even more important.

The inherited neuropathies can broadly be classified into two groups: Those in which the neuropathy is the sole or primary part of the disease (e.g., Charcot-Marie-Tooth disease, CMT) and those in which the neuropathy is part of a more generalized neurological or multisystem disorder [Table 1]. The latter group encompasses a very large group of disorders and although there have been major advances in both the diagnosis and the treatment of some of these disorders (e.g., liver transplantation for familial amyloid polyneuropathy, FAP), this review will concentrate on CMT and the related disorders of hereditary sensory and autonomic neuropathy (HSAN) and distal hereditary motor neuropathy (dHMN), as these are the commonest worldwide.

**Table 1:** Classification of the genetic neuropathies

*Neuropathies in which the neuropathy is the sole or primary part of the disorder*
Charcot-marie-tooth disease (CMT)
Hereditary neuropathy with liability to pressure palsies (HNPP)
Hereditary sensory and autonomic neuropathies/hereditary sensory neuropathies (HSAN/HSN)
Distal hereditary motor neuropathies (dHMN)
Hereditary neuralgic amyotrophy (HNA)
*Neuropathies in which the neuropathy is part of a more widespread neurological or multisystem disorder*
Familial amyloid polyneuropathy (FAP)
Disturbances of lipid metabolism (e.g., adrenoleukodystrophy)
Porphyrias
Disorders with defective DNA (e.g., ataxia telangiectasia)
Neuropathies associated with mitochondrial diseases
Neuropathies associated with hereditary ataxias
Miscellaneous

## Classification

Although many of the causative genes have been identified for CMT, a purely genetic classification is not yet possible or practical. Current classifications [Table 2] combine the traditional neurophysiological classification with the more recently identified genes and although these classifications are not entirely satisfactory they are useful to clinicians in practice. CMT is traditionally classified into two types: CMT1 (demyelinating) and CMT2 (axonal); this classification is based on upper limb (median or ulnar nerve) motor conduction velocities (MCV), with CMT1 defined as those cases where MCV is less than 38 m/s and CMT2 as those where MCV is more than 38 m/s.[1] Historically, CMT was more commonly called hereditary motor and sensory neuropathy (HMSN) but CMT is the preferred term currently. HMSNI and HMSNII are identical to CMT1 and CMT2, respectively. In the HMSN classification, severely affected infants with the demyelinating form were classified as having HMSNIII (also called congenital hypomyelinating neuropathy (CHN) or Dejerine-Sottas neuropathy (DSN)). This group of diseases was thought to be autosomal recessive (AR) but since many of these patients actually have *de novo* dominant (AD) mutations in the genes that commonly cause AD CMT1, we now use the terms CHN or DSN for this group and hence there is no CMT3. There are AR forms of both CMT1 and CMT2 and, confusingly, the AR forms of CMT1 are now called CMT4, while the much more rare AR CMT2 cases are generally referred to as AR CMT2. Table 2 is a summary of the currently known main CMT genes (space constraints do not allow all loci to be included).

**Table 2:** Classification of Charcot-Marie-Tooth disease

**Type**	**Gene/locus**	**Specific phenotype**
Autosomal dominant CMT1		
(AD CMT1)		
CMT 1A	Dup 17p (PMP22)	Classic CMT1
	PMP22 (point mutation)	Classic CMT1 / DSN / CHN / HNPP
CMT 1B	MPZ	CMT1/ DSN / CHN / intermediate / CMT2
CMT 1C	LITAF	Classic CMT1
CMT 1D	EGR2	Classic CMT1 / DSN / CHN
CMT 1	NEFL	CMT2 but can have slow MCVs in CMT1 range +/− early onset severe disease
Hereditary neuropathy with liability to pressure palsies (HNPP)		
HNPP	Del 17p (PMP-22)	Typical HNPP
	PMP-22 (point mutation)	Typical HNPP
X-linked CMT1 (CMT 1X)		
CMT 1X	GJB1	Intermediate +/− patchy MCVs / male MCVs < female
		MCVs
Autosomal recessive demyelinating (CMT4)		
CMT4A	GDAP1	CMT1 or CMT2 usually early onset and severe / vocal cord and diaphragm paralysis described / rare AD CMT2 families described
CMT4B1	MTMR2	Severe CMT1 / facial/bulbar/focally folded myelin
CMT4B2	MTMR13	Severe CMT1 / glaucoma/focally folded myelin
CMT4C	KIAA1985 (SH3TC2)	Severe CMT1 / scoliosis/cytoplasmic expansions
CMT4D (HMSNL)	NDRG1	Severe CMT1 / gypsy/deafness/tongue atrophy
CMT4E	EGR2	Classic CMT1 / DSN / CHN
CMT4F	PRX	CMT1 / more sensory/focally folded myelin
CMT4H	FGD4	CMT1
CMT4J	FIG4	CMT1
CCFDN	CTDP1	CMT1 / gypsy / cataracts / dysmorphic features
HMSN Russe	10q22-q23	CMT1
CMT1	PMP22 (point mutation)	Classic CMT1 / DSN / CHN / HNPP
CMT1	MPZ	CMT1 / DSN / CHN / intermediate / CMT2
Autosomal dominant CMT2		
(AD CMT 2)		
CMT 2A	KIF1Bb	Classic CMT2
CMT 2A	MFN 2	CMT2 / usually severe / optic atrophy
CMT 2B	RAB7	CMT2 with predominant sensory involvement and sensory complications
CMT 2C	12q23 - q24	CMT2 with vocal cord and respiratory involvement
CMT 2D	GARS	CMT2 with predominant hand wasting / weakness or dHMN-V
CMT 2E	NEFL	CMT2 but can have slow MCVs in CMT1 range +/- early onset severe disease
CMT 2F	HSP27 (HSPB1)	Classic CMT2 or dHMN-II
CMT 2G	12q12-q13.3	Classic CMT2
CMT 2L	HSP22 (HSPB8)	Classic CMT2 or dHMN-II
CMT 2	MPZ	CMT1/ DSN / CHN / intermediate / CMT2
CMT 2 (HMSNP)	3q13.1	CMT2 with proximal involvement
Autosomal recessive CMT 2		
AR CMT2A	LMNA	CMT2 proximal involvement and rapid progression described / also causes muscular dystrophy / cardiomyopathy / lipodystrophy
AR CMT2B	19q13.1-13.3	Typical CMT2
AR CMT2	GDAP1	CMT1 or CMT2 usually early onset and severe / vocal cord and diaphragm paralysis described / rare AD CMT2 families described
Dominant intermediate CMT		
(DI-CMT)		
DI-CMTA	10q24.1-25.1	Typical CMT
DI-CMTB	DNM2	Typical CMT
DI-CMTC	YARS	Typical CMT
Hereditary neuralgic amyotrophy (HNA)		

AD = autosomal dominant; AR = autosomal recessive; Dup = duplication; Del = deletion; PMP-22 = peripheral myelin protein 22; MPZ- myelin protein zero; LITAF = lipopolysaccharide-induced tumor necrosis factor; EGR2 = early growth response 2; GJB1 = Gap junction protein beta1; GDAP1 = ganglioside- induced differentiation-associated protein 1; MTMR2 = myotubularin-related protein 2; MTMR13 = myotubularin-related protein 13; SH3TC2 = SH3 domain and tetratricopeptide repeats 2, NDRG1= N-myc downstream-regulated gene 1; PRX = periaxin; CTDP1 = CTD phosphatise subunit 1; FGD4 = FYVE; RhoGEF and PH domain containing 4; FIG4 = FIG 4 homolog; KIF1Bß = kinesin family member 1B-ß; MFN2 = mitofusin 2; RAB7 = RAB7, member RAS oncogene family; GARS = glycyl-tRNA synthetase; NEFL = neurofilament, light polypeptide 68kDa; HSP 27 = heat shock 27kDa protein 1; HSP 22 = heat shock 22kDa protein 8; LMNA = lamin A/C; DMN2 = dynamin 2; YARS = tyrosyl-tRNA synthetase; SEPT9 = septin 9

Hereditary sensory and autonomic neuropathy (HSAN) describes forms of inherited neuropathies in which sensory (and, in some types, autonomic) involvement predominates [Table 3]; distal hereditary motor neuropathies (dHMN) describe purely motor forms [Table 4].

**Table 3:** Classification of the hereditary sensory and autonomic neuropathies

**Type**	**Inheritance**	**Gene/locus**	**Specific phenotype**
HSAN I (HSN1)	AD	SPTLC1	Mainly sensory, sensory complications, motor involvement variable, neuropathic pain
CMT2B	AD	RAB7	Sensorimotor, sensory complications, no pain
HSAN 1B	AD	3p22-p24	Sensory, cough, gastroesophageal reflux
HSAN II	AR	HSN2	Severe sensory complications, mutilations, onset first two decades
HSAN III	AR	IKBKAP	Familial dysautonomia or Riley-Day syndrome, prominent autonomic, absence fungiform papillae of the tongue
HSAN IV	AR	NTRK1	Congenital insensitivity to pain with anhydrosis (CIPA), severe sensory, anhydrosis, mental retardation, unmyelintated fibers mainly affected
HSAN V	AR	NTRK1	Congenital insensitivity to pain with mild anhydrosis, no mental retardation, small myelinated fibers mainly affected
HSAN V	AR	NGFB	Congenital insensitivity to pain, minimal autonomic, no mental retardation, mainly unmyelinated fibers affected
Channelopathy	AR	SCN9A	Congenital insensitivity to pain. Associated insensitivity to pain

SPLTC1 = serine palmitoyltransferase, long-chain base subunit-1; HSN2 = hereditary sensory neuropathy type II gene; IKBKAP = inhibitor of kappa light polypeptide gene enhancer in B-cells, kinase complex-associated protein; NTRK1 = neurotrophic tyrosine kinase receptor type 1; NGFB = nerve growth factor beta polypeptide; SCN9A = sodium channel, voltagegated, type IX, alpha subunit

**Table 4:** Classification of the distal hereditary motor neuropathies

**Type**	**Inheritance**	**Gene/locus**	**Specific phenotype**
HMN I	AD	unknown	juvenile-onset dHMN
HMN II	AD	HSP27 (HSPB1)	Adult-onset typical dHMN/+/− minor sensory (CMT4F)
HMN II	AD	HSP22 (HSPB8)	Adult-onset typical dHMN/+/− minor sensory (CMT2L)
HMN III	AR	11q13	Early-onset, slowly progressive
HMN IV	AR	11q13	Juvenile-onset, diaphragmatic involvement
HMN V	AD	GARS	Upper limb onset, slowly progressive/CMT2D
HMN V	AD	BSCL2	Upper limb onset, +/−spasticity lower limbs/Silver syndrome
HMN VI	AR	IGHMBP2	Spinal muscle atrophy with respiratory distress (SMARD1), infantile-onset respiratory distress
HMN VIIA	AD	2q14	Adult-onset, vocal cord paralysis
HMN VIIB	AD	DCTN1	Adult-onset/vocal cord paralysis/facial weakness
HMN/ALS4	AD	SETX	Early-onset, pyramidal signs
HMN-J	AR	9p21.1-p12	Juvenile-onset, pyramidal features, Jerash
Congenital distal SMA	AD	12q23-12q24	Antenatal-onset, arthrogryposis

HSP 27 = heat shock 27kDa protein 1; HSP 22 = heat shock 22kDa protein 8; GARS = glycyl-tRNA synthetase; BSCL2 = Berardinelli-Seip congenital lipodystrophy 2 (Seipin); IGHMBP2 = immunoglobulin mu binding protein 2; DCTN1 = dynactin1; SETX = sentaxin

This classification system is not perfect, and as we identify the causative genes it is clear that there is overlap between various types of CMT / HSAN / dHMN. For example, HSAN1 and CMT2B are clinically difficult to differentiate despite being caused by mutations in different genes, and certain genes for dHMN (*GARS*, *HSP27*, and *HSP22*) also cause axonal CMT.

## Is the neuropathy genetic?

The first step in making the diagnosis of CMT is to determine whether the patient has a genetic neuropathy. Sometimes the answer is clear; for example, when there is an affected parent and child, either autosomal dominant (AD) or X-linked (if there is no definite male- to-male transmission) inheritance is probable, or when there are affected siblings from a consanguineous marriage, autosomal recessive (AR) inheritance is likely. X-linked inheritance should always be kept in mind unless there is unequivocal evidence of male-to-male transmission, especially as families are often not large enough to confidently rule this possibility out. In other patients, recognizing CMT can be more difficult. There may be no family history or families may be small and extensive family histories may not be available. Clinical features that may help the clinician decide whether the neuropathy is genetic include presentations in infancy; a long, slow progression; the presence of foot deformities; and, in adults, the lack of positive sensory symptoms (e.g., dysaesthesias or paresthesias) in the presence of clear sensory signs. If the clinical features raise the possibility, it is important not to rule out an inherited neuropathy, even in the absence of a family history.

## CMT

CMT is a common condition, with an estimated prevalence of 1 in 2500 in the general population.[2] This figure may be an underestimate as the true prevalence figures for CMT2 are not available at present since 75% of the causative genes are not yet known. As stated earlier, CMT is usually classified as either demyelinating (CMT1) or axonal (CMT2) [Table 2]. The MCVs in the upper extremities are usually uniformly slow in demyelinating CMT, whereas there is often patchy, asymmetric slowing, with conduction block and dispersion, in acquired demyelinating neuropathies (e.g., acute or chronic inflammatory demyelinating polyradiculoneuropathy).[3,4] This can be a helpful pointer diagnostically. Intermediate forms of CMT, with median or ulnar nerve MCVs between 25-45 m/s, are also present and may help direct the genetic diagnosis (see below). Sporadic cases of CMT occur often and are usually found to have mutations in the common AD genes (autosomal dominant or *de novo* dominant) or in the AR genes. The occurrence of *de novo* dominant mutations explains how the parents of these patients are found to be normal (clinically, neurophysiologically, and genetically). These cases are, however, very important to diagnose because they are *de novo* dominant, meaning that the children of these patients have a 50% risk of inheriting the mutant gene unlike the children of a patient with AR mutations. In most UK / North European and US populations, about 90% of cases of CMT are either AD or X-linked, whereas in countries with a higher rate of consanguineous marriages, AR CMT can account for up to 40%.[5] The diagnostic approach will therefore vary in specific countries and in specific ethnic groups. In all populations, CMT1 is consistently reported to be more common than CMT2 but, as previously stated, 75% of the genes have yet to be identified for CMT2 and therefore the true prevalence of CMT2 is unknown.

## AD CMT1: Autosomal dominant demyelinating CMT

**Figure 1:**
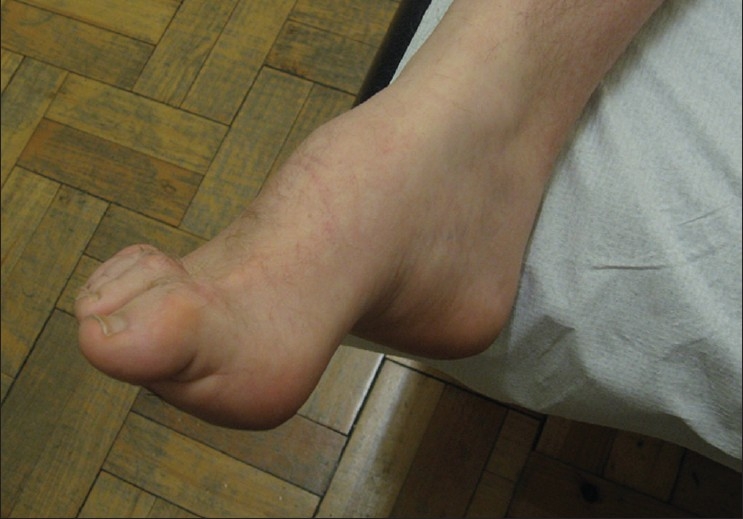
Typical foot of patient with CMT, showing pes cavus and clawed toes

**Figure 2:**
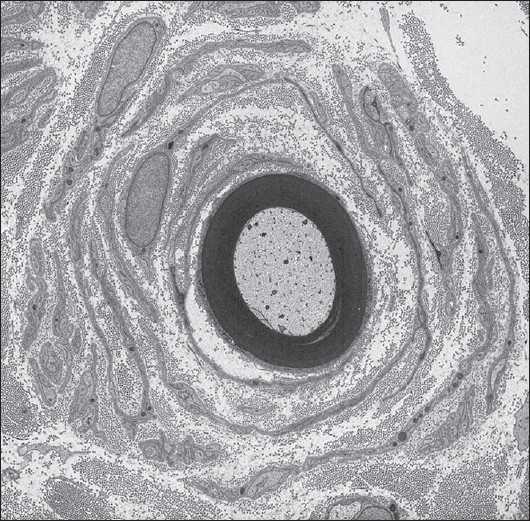
Electron micrograph of a sural nerve biopsy from a patient with CMT1A secondary to the chromosome 17 duplication, showing a classical onion bulb

AD CMT1 is the most common form of CMT in most populations. Patients usually present with a ‘classical CMT phenotype’ that includes lower limb motor symptoms (e.g., difficulty in walking) beginning in the first two decades, along with distal weakness, atrophy, sensory loss, hyporeflexia, and foot deformity, e.g., pes cavus [Figure 1]. It is unusual for patients to present with sensory symptoms. It is also often difficult to accurately ascertain the age of onset as the symptoms in the first decade of life may be nonspecific (e.g., poor at sports, last in races, clumsiness). Patients usually have normal life spans, frequently need ankle-foot orthotics (AFOs), and rarely require wheelchairs for routine ambulation. The upper limbs are involved later than the lower limbs and it is very common for the patients not to complain of hand weakness (although it is detectable on examination) unless asked specific questions (e.g., do you have difficulty doing your buttons and zips or unscrewing the tops of bottles?). Median and ulnar MCVs are below 38 m/s and the sensory action potentials (SAPs) are either reduced or absent. Nerve biopsy demonstrates demyelination and onion bulb formation [Figure 2]. However, biopsies are no longer necessary to make the diagnosis.

**Figure 3:**
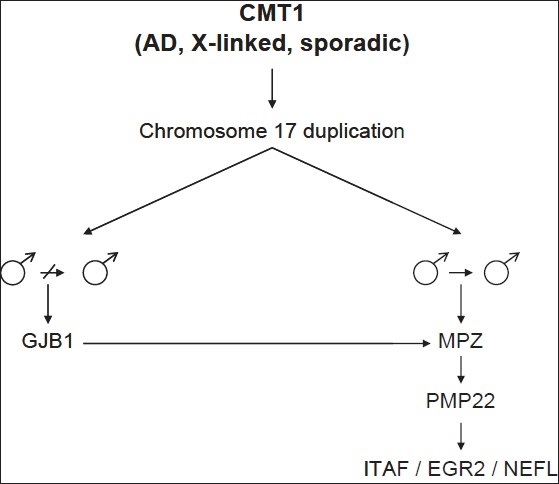
Algorithm for molecular diagnosis of AD and X-linked Charcot-Marie-Tooth disease 1 (CMT1)

The approach to the genetic diagnosis of AD CMT1 requires careful clinical examination, neurophysiological testing, and an appreciation of the frequency with which a particular gene causes AD CMT1 [Table 2, Figure 3]. As a general rule, most AD CMT1 patients have a classical phenotype and the approach to the genetic diagnosis is therefore based primarily on the frequency of the underlying genetic causes. Classic CMT phenotypes and MCV less than 38 m/s (commonly around 20 m/s) are strongly suggestive of CMT1A, which is caused by a 1.4- Mb duplication on 17p11.2.[6,7] Sporadic cases occur in about 10% of CMT1A cases. Thus the lack of family history does not exclude CMT1A. In European populations CMT1A accounts for 70% of all CMT1 cases.[8] This frequency may be less in populations with a high rate of consanguinity, where AR CMT1 cases may have an increased frequency. The 1.4-Mb duplication contains the *PMP22* gene, meaning that patients with this form of CMT1A have three copies of the *PMP22* gene and this increased dose of *PMP22* is the cause of this type of CMT1A. Mutations in the *PMP22* gene can also cause CMT1, but with a wider spectrum of phenotypes, including classical CMT1A but also the more severe DSN. In addition, certain mutations can cause hereditary neuropathy with liability to pressure palsies (HNPP) (see below).

CMT1B caused by mutations in *myelin protein zero (MPZ)* comprise about 10% of AD CMT1 and are therefore the second commonest type. Patients can present with the classical CMT1 phenotype but are more likely to have either a more severe early-onset form of CMT with MCV < 10 m/s or a late-onset form with median MCVs in the axonal range.[9]

**Figure 4:**
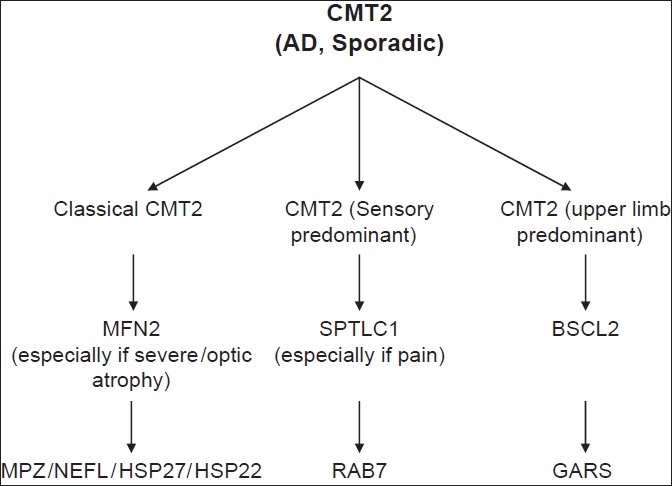
Algorithm for molecular diagnosis of AD Charcot-Marie-Tooth disease 2 (CMT2)

*EGR2* and *SIMPLE* mutations are rare (< 1%) causes of AD CMT1.[10,11] Patients with *EGR2* mutations usually present with the more severe DSN phenotype and patients with *SIMPLE* mutations frequently resemble those with CMT1A. Mutations in *NEFL* were originally described as a cause of CMT2,[12] but some patients have MCVs in the demyelinating range[13] in spite of the fact that the gene is expressed in neurons but not Schwann cells.

## HNPP

HNPP is an AD neuropathy usually caused by a deletion of the same 1.4-Mb portion of chromosome 17 that is duplicated in CMT1A.[14] Therefore, patients have only one copy of *PMP22*, and this decreased dose of *PMP22* is the cause of HNPP. Nonsense or frameshift mutations that truncate *PMP22* and effectively cause a loss of function of one copy of *PMP22* are rare causes of HNPP. Patients typically present with transient, recurrent episodes of focal weakness or sensory loss, usually precipitated by pressure in the distribution of individual nerves or plexuses.[15] Peripheral nerves that are susceptible to pressure along their routes are most commonly involved (e.g., the common peroneal nerve at the fibular head or the ulnar nerve at the elbow). Nerve conduction studies often show focal areas of slowing around sites subject to compression.[16] Screening patients with isolated pressure palsies (such as carpal tunnel syndrome) for HNPP is not warranted as to date all patients shown to have the genetic abnormality have had widespread nerve conduction abnormalities, even if they have involvement of only one nerve clinically.

## X-linked CMT1

X-linked CMT1 is the second commonest form of CMT and is caused by mutations in the *gap junction protein beta 1 (GJB1) gene* encoding connexin 32 (Cx32).[17] As expected with an X-linked disorder, males are usually more severely affected than females. Females may be very mildly affected and even asymptomatic though signs can usually be elicited. Cx32 is expressed in myelinating Schwann cells, not in neurons.[18] Nerve conduction is commonly slower in males (demyelinating CMT1 range) than in females, who are usually in the axonal CMT range; however, in both males and females the NCVs can often be in the intermediate range (25-40 m/s).[19] Occasional CMT1X patients have asymmetric MCVs reminiscent of CIDP.[20] Although oligodendrocytes also express Cx32, the CNS is most often only occasionally involved in CMT1X and usually asymptomatically (e.g., the only abnormalaties present may be extensor plantars, mild deafness, abnormal brainstem evoked potentials).[21] However, occasionally transient severe CNS involvement characterized by ataxia and dysarthria has been described.[22] There are over 300 causative *GJB1* mutations (http://www.molgen.ua.ac.be/CMTMutations/default.cfm). Unlike distinct *PMP22* and *MPZ* mutations, virtually all *GJB1* mutations have similar age-related phenotypes that resemble those in which the gene is entirely deleted.[23]

## AD CMT2: Autosomal dominant axonal CMT

AD CMT2 can be difficult to distinguish from an idiopathic axonal neuropathy when there is no family history. This is particularly a problem in older patients presenting with an incidental idiopathic axonal neuropathy that is mild and long-standing and with no family history or no family members available for examination. Eight causative genes have been identified [Table 2], which account for about 25% of all AD CMT2 cases.

Unlike AD CMT1, AD CMT2 can be subdivided into three distinct phenotypes [Figure 2]. The first and most common one is the 'classical CMT' phenotype. This is indistinguishable from the classical AD CMT1 phenotype until neurophysiological testing is done, although much later ages of onset may sometimes be seen in AD CMT2 than in AD CMT1. Nerve biopsies are rarely helpful diagnostically, showing only an axonal neuropathy without any specific diagnostic features. Mutations in *mitofusin 2 (MFN2)* cause CMT2A[24] which represents about 20% of all AD CMT2 cases. Although *MFN2* is usually the first gene screened in classical AD CMT2, CMT2A patients with *MFN2* mutations generally have a severe phenotype that may severely impair them in childhood. Twenty percent of *MFN2* mutations are *de novo.* Occasional CMT2A patients also have optic atrophy (these cases were termed HMSN VI in previous classifications)[25] brisk reflexes, and / or minor white matter changes on brain MRI.[26] Mutations in *MPZ* and *NEFL* can also cause the classical AD CMT2 phenotype as mentioned above. Small heat shock protein genes *HSP27* (HSPB1) and *HSP22* (HSPB8) are rare causes of the classical AD CMT2 phenotype, but such cases usually have minimal sensory involvement. These two genes can also cause a purely motor phenotype, dHMN type II.[27] A homozygous mutation in *HSP27* has also recently been described to cause AR CMT2.[28]

Profound sensory impairment, often including ‘ulceromutilations,’ characterizes the second AD CMT2 phenotype.[29] Unlike the classical CMT phenotype, these patients present with lack of sensation and the complications of sensory loss, including ulcerations, osteomyelitis, and amputations. Motor involvement is present but is usually less than the sensory involvement. The causative gene is *RAB7* and patients are classified as having CMT2B.[30] CMT2B patients are difficult to distinguish from those with HSAN1 caused by mutations in the *SPTLC1* gene although, unusually for hereditary neuropathy patients, the patients with *SPTLC1* mutations often have neuropathic pain.[31,32] If patients present with prominent sensory features, both the *RAB7* and *SPTLC1* genes should be initially screened.

The third phenotype described in AD CMT2 is exemplified in patients with CMT2D. Unlike the classical length-dependent CMT patients, who present with lower limb involvement first, these patients present with atrophy and weakness of the small muscles of the hand (which can be unilateral and may be misdiagnosed as thoracic outlet syndrome); much later, there is involvement of the distal lower limb muscles. CMT2D is caused by mutations in *GARS.*[33] Some patients have no sensory involvement and have been classified as dHMN type V, an allelic condition. The dHMN V/CMT2D phenotype has subsequently been shown to be more commonly due to mutations in the *BSCL2* gene,[34] which usually causes Silver syndrome (spastic legs and distal amyotrophy of the upper limbs) but can present (in 33% of cases) with just amyotrophy of the upper limbs. Therefore, in patients presenting with AD CMT2 and predominant involvement of the small hand muscles, *BSCL2* and *GARS* should be screened first.

## Autosomal recessive CMT

In most communities AR CMT is less common than AD CMT. AR CMT1 is also classified as CMT4, whereas AR CMT2 is usually just called AR CMT2. Thirteen genes have been identified that cause AR CMT4 (including three genes - *PMP22, MPZ*, and *EGR2* - that also cause AD CMT1) [Table 2]. No one algorithm is suitable for the evaluation of CMT4, but there are simple clinical rules that can be helpful in diagnosis. Usually CMT4 cases have early, infantile onset (DSN or CHN) and are severe. Weakness often progresses to involve proximal muscles and may result in early loss of ambulation. Unlike most cases of AD CMT1, nerve biopsies can be useful in certain cases because specific features make a particular genetic diagnosis more likely (see below). Certain clinical features and the patient's ethnic background may help direct genetic diagnosis [Table 2].

In assessing AR CMT patients, particular points to consider include:

Identifying demyelination by MCV can be difficult in CMT4 because motor and sensory amplitudes are often unobtainable at routine recording sites as the patients are too severely affected. Conduction studies of nerves innervating proximal muscles may be necessary to identify slow MCV. Nerve biopsies are sometimes done in these circumstances to help clarify the type of CMT.

Nerve biopsies showing focally folded myelin are characteristic of CMT4B1 (*MTMR2* mutations) and CMT4B2 (*MTMR13* mutations) but can also be seen with *MPZ* mutations and in CMT4F secondary to periaxin mutations.

Severe and early scoliosis may be seen with CMT4C due to mutations in the *KIAA1985* gene. Characteristic nerve biopsy features including basal membrane onion bulbs and multiple cytoplasmic processes of the Schwann cells ensheathing unmyelinated axons are also seen with KIAA1985 mutations.[35]

Three forms of AR CMT are largely confined to patients of Balkan gypsy origin. CMT4D secondary to *NDRG1* mutations is characterized by a demyelinating neuropathy with a high prevalence of deafness. Tongue atrophy has also been described. CCFDN (congenital cataract, facial dysmorphism, and neuropathy syndrome) secondary to *CTDP1* mutations is also found in gypsies. A third form of AR CMT1 has been described in Balkan gypsies (HMSN Russe) but the gene has yet to be identified.

Predominant sensory involvement and variable phenotypes are characteristic of CMT4F (periaxin mutations).

AR CMT2 is very rare compared to AD CMT2. Only two causative genes have been identified (*LMNA* and *GDAP1*) [Table 2]. *GDAP1* mutations are also the commonest cause of CMT4, a demyelinating form of AR CMT and therefore *GDAP1* is often the first gene screened in most forms of AR CMT. Most patients with mutations in lamin A/C (*LMNA*)[36] present in the second decade with a severe CMT phenotype, including proximal muscle involvement, although some have a milder phenotype. Lamin A/C mutations have been associated with a wide spectrum of other phenotypes, including Emery-Dreifuss muscular dystrophy, cardiomyopathy, and Dunnigan-type familial partial lipodystrophy.

## Intermediate CMT

Certain forms of CMT characteristically present with MCVs in the intermediate range (25–45 m/s) These include dominant intermediate (DI)-CMTB caused by *DNM2* mutations;[37] DI-CMTC caused by *YARS* mutations;[38] and DI-CMTA, in which only linkage has been identified at 10q24.1-25.1. In addition, as discussed above, patients with CMT1X (*GJB 1* mutations), CMT2E (*NEFL* mutations), late-onset CMT1B (*MPZ* mutations), and CMT4A (*GDAP1* mutation) often present with intermediate MCVs.

## Hereditary sensory and autonomic neuropathy

The hereditary sensory and autonomic neuropathies are rarer than CMT but many of the genes have been identified [Table 3]. Autonomic abnormalities are often minimal in these conditions [hence certain forms are sometimes referred to as hereditary sensory neuropathies (HSN)] and motor involvement can be present. The sensory loss can lead to severe complications, including recurrent injuries, ulcerations, osteomyelitis, and amputations. The commonest AD form is HSAN1 (or HSN1) caused by *SPTLC1* mutations. Patients usually present in the second decade with distal lower limb sensory loss and many have neuropathic pain as described above. Motor involvement can be significant, especially later in the disease course, making it difficult to distinguish this disorder from certain forms of CMT2. MCVs can be in the demyelinating range, with males being more severely affected than females.[39] As discussed above, this disease is very difficult to differentiate from CMT2B secondary to *RAB7* mutations although the lancinating pain in patients with *SPTLC1* mutations can be a useful guide to this diagnosis.

HSAN II is an early-onset, autosomal recessive, severe sensory neuropathy with prominent sensory complications; it is due to mutations in the *HSN2* gene.

HSAN 111 (Riley-Day syndrome) is an AR neuropathy seen in Ashkenazi Jews and is characterized by mainly autonomic involvement, though it also involves the peripheral nervous system, particularly the sensory nerves. The causative gene is the *IKBKAP* gene. The predominant autonomic features make this type relatively easy to distinguish from the other forms of HSAN.

HSAN IV and V are both AR neuropathies characterized by congenital insensitivity to pain. HSAN IV [also called congenital insensitivity to pain with anhidrosis (CIPA)] presents with a severe sensory neuropathy, anhidrosis, and mental retardation and is due to mutations in the *NTRK1* gene. HSAN V is similar, though without the mental retardation or significant anhidrosis, and has been shown to result from mutations in *NTRK1* and also *NGFB*.

Recently, the identification of homozygous mutations in the *SCN9A* gene as a rare cause of congenital insensitivity to pain[40] has been of great interest because heterozygous mutations in the same gene cause hereditary erythermalgia[41] and paroxysmal extreme pain disorders.[42] The finding of mutations in a channelopathy gene in these painful neuropathies will hopefully expand the potential therapeutic possibilities.

## Distal hereditary motor neuropathies

The dHMNs are a complex group of disorders [Table 4] that typically present with length-dependent weakness and no sensory loss. They can be indistinguishable from certain forms of CMT until neurophysiology confirms normal sensory action potentials, as some CMT patients have not only no sensory symptoms but also no sensory signs. DHMN II is the classic form of AD dHMN and is due to mutations in the *HSP27* and *HSP22* genes, which also cause CMT2F and CMT2L. There are many other forms, but the genes are not known for all types. The main types, other then dHMN II, are summarized below:

Mutations in *GARS* and *BSCL2* cause dHMN V (also CMT2D), which is characterized by onset in the upper limbs as described above.

DHMN VI, an unusual severe AR form of dHMN, presents in infancy with respiratory and distal limb involvement (called spinal muscle atrophy with respiratory distress type 1). This is due to mutations in *IGHMBP2*.

Mutations in dynactin (*DCTN1*) cause one form of dHMN type VII, which is characterized by vocal cord paralysis and progressive weakness and atrophy of the face, hands and legs.

Missense mutations in senataxin (*SETX*) can cause a form of dHMN with pyramidal features whereas nonsense mutations in the same gene cause AR ataxia oculomotor apraxia type 2 (AOA2).

## Conclusion

The hereditary neuropathies are clinically and genetically heterogeneous. Although the area is complex and the number of causative genes is increasing rapidly, algorithms to guide genetic testing for the more common AD disorders and careful phenotyping for the rare AR disorders should help diagnosis. Neurophysiological testing remains crucial in aiding diagnosis. One of the major issues throughout the world is the lack of availability of testing for the rarer genes. It cannot be emphasized how important an accurate genetic diagnosis is for these patients. There are obvious benefits; for example, diagnostic testing for other family members can be done, accurate genetic counseling and prognostication becomes possible, as also predictive testing, antenatal testing, and preimplantation diagnosis. Furthermore, an accurate diagnosis means that patients need not have invasive diagnostic tests, e.g., nerve biopsies and lumbar punctures. In certain situations where inflammatory neuropathies are being considered, a genetic diagnosis can also prevent a trial of potentially dangerous immunosuppressive therapy. Finally, the era of specific treatment for genetic neuropathies is now here. The first therapeutic trials are ongoing (ascorbic acid for CMT1A) and because any treatments developed are likely to be gene specific, the case for an accurate genetic diagnosis will become even more convincing.
